# Assessment of Availability of Tracer Drugs and Basic Diagnostics at Public Primary Health Care Facilities in Ethiopia During COVID-19 Pandemic

**DOI:** 10.4314/ejhs.v33i2.7S

**Published:** 2023-10

**Authors:** Abdurezak Umer, Hussen Mohammed, Bekele Yazie, Dessie Abebaw Angaw, Tajebew Zayede Gonete, Berhanu Fikadie Endehabtu, Binyam Tilahun, Meskerem Jisso, Alemu Tamiso, Netsanet Abera Assesfa, Akalewold Alemayehu, Rekiku Fikre, Biru Abdissa Mizana, Kassahun Dessie, Habtamu Sime, Muluemebet Abera, Mohammed Mecha, Elias Ali Yesuf, Kassu Ketema Gurmu, Mesfin Kebede

**Affiliations:** 1 Dire Dawa University, College of Medicine and Health Sciences, Ethiopia; 2 University of Gonder, College of Medicine and Health Science, Institute of Public Health, Gonder, Ethiopia; 3 Hawassa University, College of Medicine and Health Sciences, Hawassa, Ethiopia; 4 Jimma University, Institute of Health, Jimma, Ethiopia; 5 World Health Organization-Ethiopia, Health System Strengthening Unit, Addis Ababa, Ethiopia

**Keywords:** tracer drugs, basic diagnostics, availability, primary health care units, Ethiopia

## Abstract

**Background:**

The emergence of COVID-19 pandemic has disrupted the supply chain and stock of medicines and drugs across the globe. Tracer drugs are essential medicines that address the population's priority health problems. Thus, this study aimed to assess availability of tracer drugs and basic diagnostics at public primary health care facilities in Ethiopia.

**Methods:**

Facility based cross-sectional study was employed in four regions and one city administration. The primary health care units (PHCUs) were purposively selected in consultation with respective regional health bureaus. Finally, 16 hospitals, 92 health centers and 344 health posts were included. This study adopted WHO's tool that was being used to rapidly assess the capacity of health facilities to maintain the provision of essential health services during the COVID-19. Descriptive analysis was done using frequency and percentage, and results were presented.

**Results:**

The overall mean availability of tracer drugs in PHCUs was 77.6%. Only 2.8% of PHCUs have all tracer drugs. The mean availability of basic diagnostic at national level was 86.6% in PHUs except health posts where it was less. Health facilities with all basic diagnostic services was 53.7%. Of the total 344 health posts assessed, 71% were providing diagnostic testing for malaria using either laboratory equipment or rapid diagnostic test (RDT) while 43% provide urine test for the pregnancy.

**Conclusion:**

This study shows availability of all tracer drugs in PHCUs in Ethiopia was extremely low. There was regional variation in availability of tracer drugs and basic diagnostics. It is very crucial to increase availability of tracer drugs and diagnostics. Drugs and diagnostic materials should be supplied according to the capacity and location of health facilities.

## Introduction

Coronavirus (Covid-19) disease was declared a global pandemic by the World Health Organization in March 2020. The virus has caused overwhelmed devastation all over the world since it originated in Wuhan, China, in December 2019(1). Globally, over 515.3 million confirmed COVID-19 cases reported, affecting over 226 countries, regions, and territories. As of May 5, 2022, the pandemic has afflicted all African countries, resulting in over 11.5 million cases of which 470,647 were in Ethiopia (2).

The emergence of COVID-19 has affected the economy at worldwide by directly affecting the production in key countries that are sole manufacturers of raw materials, intermediate producers, and consumer goods, thereby creating supply chain and market disruption, and by its financial impact on firms and the financial markets including availability of essential drugs and basic diagnostics(3).The cost of the pandemic has also been disastrous on human health, social networks, and economic subsistence in many societies. African countries, in particular, lack the capacity, performance, and preparedness to respond to outbreaks while maintaining routine health services (4–6).

Tracer drugs are essential medicines that address the population's priority health problems and their treatments. As a result of the surge in the pandemic, which led to the inevitable lockdown of the economy across affected countries, there has been a noticeable decrease in production and exportation of raw materials as well as finished products (drugs) across several countries. These greatly affected the ease of access to these medicines by the consumers who need them either for treating acute ailments or for the management of chronic diseases. Most developing countries including Ethiopia are in their early stages of pharmaceutical development; thus, they rely on importation of drugs, raw materials, and equipment from countries outside the region, notably India and China(7–9).

To provide consistent, high-quality care, and to manage the prioritized community health problems, the tracer drugs should be available and accessible at all times at healthcare institutions. Tracer drugs availability and access are core indicators of supply chain performance. It is a proxy indicator of a health program's ability to meet clients' needs with a full range of products and services. Furthermore, the country has faced challenges such as a nationwide shortage of essential pharmaceuticals, inadequate cold chain infrastructure, limited coverage of diagnostic and treatment facilities, ineffective stock management, and a lengthy procurement process(10–13).

Thus, it has been a major concern in the country health sector transformation plan that envisions improving healthcare product management systems to ensure uninterrupted availability and accessibility to address health problems. The Government of Ethiopia has taken public health and political measures as part of worldwide efforts to contain the virus from spreading (14). A state of emergency was declared, and border closures, as well as travel and trade restrictions between countries, resulted in a considerable reduction in the number of essential medicines and diagnostic materials, making it difficult for healthcare facilities to get the products they need(14–16). Thus, this study aimed to assess availability of tracer drugs and basic diagnostics at public primary health care facilities in Ethiopia in the context of the coronavirus pandemic.

## Materials and Methods

**Study design, setting and period:** Between October and December 2021, a facility based cross-sectional study was conducted in four regions and one city administration, namely Amhara, Oromia, Sidama and Southern Nations Nationalities and Peoples (SNNP) regions and Dire Dawa Administration.

**Sample size and sampling technique:** The selection of PHCUs was made in consultation with respective regional health bureaus, the World Health Organization proposed intervention plan following baseline assessment; hence, attempts were made to include facilities with all forms of performance: highest, medium and lowest performance based on their last six months performance assessment.

All primary hospitals that found in the selected Woredas (districts) and selected PHCUs with all satellite health posts under the selected health centers were included in the study. Overall, the assessment was undertaken in 452 health facilities (16 primary hospitals, 92 health centers and 344 health posts).

**Data Collection Tool and Procedure:** This study adopted WHO's “*continuity of essential health services: facility assessment tool*” that can be used to rapidly assess the capacity of health facilities to maintain the provision of essential health services during the COVID-19 pandemic and continuity after the recovery. The assessment tool covers key aspects of essential health services including sexual and reproductive health services (8).

The standard questionnaire was converted to electronic form using *KoboCollect* form. Data collectors and supervisors having experience of electronic data collection using *KoboCollect* were recruited by consortium universities: Dire Dawa University for the city administration setting study, University of Gondar for selected study settings in Amhara, Jimma University for study settings in Oromia and Hawassa University for the two regions, namely Sidama and SNNP studies.

**Data quality Assurance:** A two days training was provided for data collectors and supervisors about the purpose of the study and the content of the tools prior to the data collection. Pre-test was also undertaken prior to the actual data collection. The *KoboCollect* form was prepared with strict rules (on relevance during e-tool development) to assure the consistency of entry across the questions and legal value entries for responses. The research team and supervisors closely followed the process. The completed forms were downloaded on daily basis 2.to monitor the progress as well as the completeness of the form. Feedback was also given on the completed forms for the data collectors before the next data collection day.

**Data analysis:** All data collectors sent the completed forms to the prepared data storage site at *KoboCollect server*. The entered data were downloaded and exported to SPSS for further cleaning and analysis. Descriptive analysis was done using frequency and percentage. Results were presented using tables and figures. so that interview data and data represented in reports were not attributed to individuals.

**Ethical clearance and consent to participate:** Ethical clearance was granted by the institutional review boards and ethics committees of, college of medicine and health sciences of Dire Dawa University, Hawassa University, Jimma University and University of Gonder. Official permission letters from the respective regional health bureau were secured to PHCUs (zonal health departments, woreda health offices and health facilities). Informed oral and written consents were ensured for all participants by informing the aim of the assessment and requesting for their willingness to participate or not. To this end, information sheets were distributed to all interviewee participants such as health posts, heads of PHCUs and RHBs staff. The interviewees were given an account of the aims of the project as a whole and the scope of the study specifically. It was made clear that participants were free to stop the interview at any time and not obligated to answer questions they not interested. Confidentiality was maintained thoroughly, interviews were anonymised

## Results

A total of 452 PHCUs from 4 regions and 1 city administration were assessed on the availability of tracer drugs and diagnostics. From the total PHCUs assessed, 3.5% were primary hospitals, 20.4% were health centers, and 76.1% were health posts, as indicated on the (table 1.)

**Table 1:** Distribution of primary health care units selected from four regions and one city administration

Types of PHCUs	Primary Hospital N (%)	Health Center N (%)	Health Post N (%)	Total N (%)
Dire Dawa	-	15 (16.3)	33 (9.6)	47 (10.4)
SNNP*	3	23 (25.0)	139 (40.4)	165 (36.5)
Sidama	3	12 (13.0)	42 (12.2)	57 (12.6)
Amhara	7	10 (10.9)	66 (19.1)	83 (18.4)
Oromia	3	32 (34.8)	64 (18.6)	99 (21.9)
Total	**16**	**92**	**344**	**452**

* SNNP: Southern Nations Nationalities and Peoples

**Figure 1:**
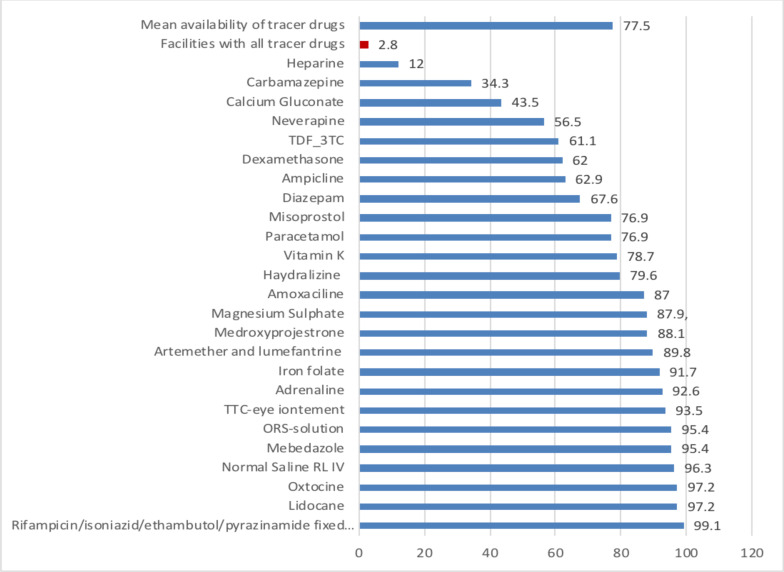
Percentages of tracer drugs availability in PHCUs excluding health posts during COVID-19 pandemic in the selected regions, Ethiopia, 2021 (N=108)

The availability of tracer drugs and diagnostics were measured using 24 drugs and 8 diagnostics kits and tools for primary hospitals, health centers and health posts. The result indicated that only 2.8% of the health facilities excluding health posts have had all tracer drugs, almost all hospitals have had Amoxicillin, about 88.9% of hospitals had Artemether and lumefantrine, and Rifampicin/isoniazid/ethambutol/pyrazinamide fixed dose combination, 77.8% of hospitals had also Nevirapine Suspension and 83.3% TDF-3TC-DTG fixed dose combination. There were evident variations among hospitals in the availability of essential medicines. Some drugs, like Carbamazepine, Hydralazine, Nevirapine Suspension and Magnesium Sulfate were not available currently in some hospitals (Figure 1).

**Figure 2:**
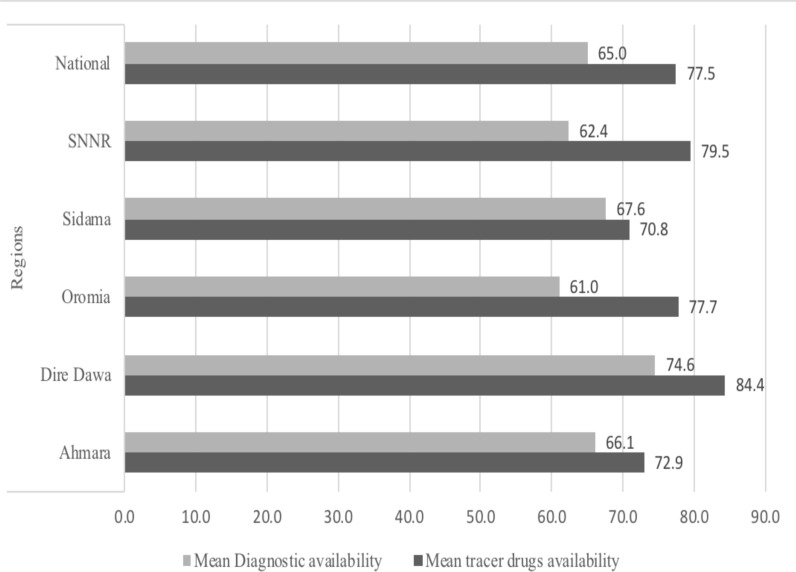
Percentages of tracer drugs and diagnostics availability by regions in PHCUs excluding health posts during COVID-19 pandemic in the selected regions, Ethiopia, 2021 (N=108)

The mean availability of essential tracer items in PHCUs excluding health posts was 77.5%. Overall, the mean availability of 24 tracer drugs was above 50% across all regions ranging from the highest percentage (84.4%) of mean tracer drugs availability among PHCUs reported from Dire Dawa City Administration to the lowest reported from facilities in Sidama region (70.8%) (Figure 2).

**Figure 3:**
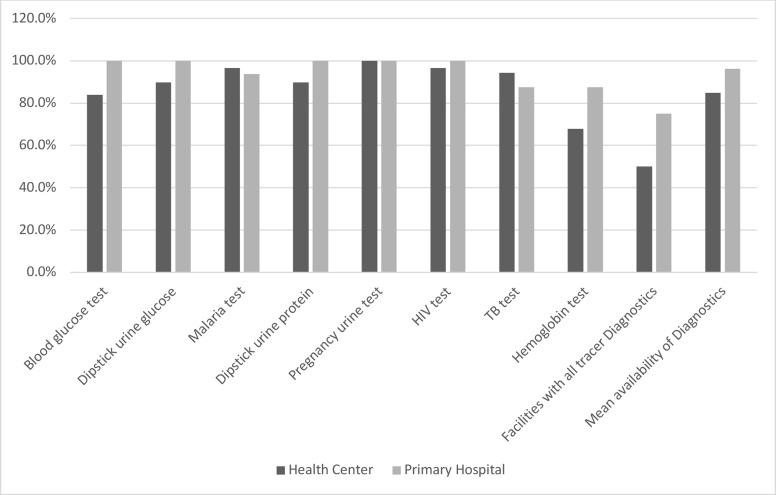
Percentages of diagnostics availability in PHCUs excluding health posts during COVID-19 pandemic in the selected regions, Ethiopia, 2021 (N=108)

Concerning essential diagnostics service availability by both facility type, different availability figures were observed. The mean availability of essential diagnostics items was higher in primary hospitals (96.1%) than in health centers (84.9%). Moreover, half of the health centers and 75% of primary hospitals reported all diagnostics. Majority of health centers had the capacity to deliver the following diagnostic tests: HIV test (RDT or ELISA), blood glucose test, malaria test (RDT or smear), urine pregnancy test, urine dipstick for protein, and urine dipstick for glucose. Comparatively only 67.8% health centers had hemoglobin tests. As Figure 3 shows almost all of primary hospitals had the capacity to provide those tests in their facilities.

Similarly, the health posts in the selected regions were assessed on the availability of tracer drugs and diagnostics. Paracetamol, TTC eye ointment, Rifampicin/isoniazid/ethambutol/pyrazinamide fixed dose combination, length/height board, and blood pressure (BP) apparatus were not available in 59.2%, 63.6%, 78.9%, 58.4%, and 55.4% of health posts in Ethiopia, respectively. Regarding the availability of diagnostic services, 244(70.9%) health posts provided diagnostic testing for malaria using RDTs while 147(42.7%) of them provided urine test for the pregnancy (Table 2).

**Table 2:** Availability of selected tracer drugs at health posts in Ethiopia during COVID-19 pandemic

Tracer drugs	No	%
Artemether and lumefantrine combination	194	56.9
Rifampicin/isoniazid/ethambutol/pyrazinamide fixed dose combination	72	21.1
Medroxy progesterone injection	270	79.2
Paracetamol	139	40.8
Zinc	141	41.3
Amoxicillin	204	59.8
TTC eye ointment	124	36.4
Iron folate	288	84.5
Mebendazole	265	77.7
Oral rehydration salts (ORS)	274	80.4

## Discussion

Access to essential medicines and supplies is basic to a well-functioning health care delivery system. Essential medicines are those that satisfy the priority healthcare needs of the population. Overall mean availability of tracers' drug was 77.6%.

It was higher than that was reported by the service availability and readiness assessment survey (SARA) in Ethiopia. Besides, only 2.8% of PHCUs have had all tracer drugs. This might be due to variation of some tracer drugs included in this study.

The mean availability of basic diagnostics at national level was 86.6% in PHCUs except health posts which was higher than SARA study conducted in Ethiopia (18). This might be due to the number of health facilities included in the studies. Other possible reason could be the efforts exerted by government and stakeholder in restoring and maintaining essential health services affected by the COVID-19 pandemic.

Three fourth of health posts reported providing diagnostic testing for malaria either laboratory equipment or RDT. But it was lower than a study conducted in Ethiopia where fifty-three (53%) (18)of the health posts reported the availability of malaria diagnosis test. This might be due to the study area difference and sample size. The previous study was conducted before the COVID-19 pandemic while this study was done during the pandemic. Similarly, only 147 (43%) of health posts were providing urine test for pregnancy, and the rest had no functional lab and items required for the test at the time of data collection. This finding is lower than a study (18). This might be due to lack of integration of sexual and reproductive health service and stock out of the diagnostic test during COVID-19 pandemic.

This study shows that availability of all tracer drugs in PHCUs in Ethiopia was low. There was regional variation in availability of tracer drug and basic diagnostics. It is very crucial to increase availability of tracer drugs and diagnostics. Drugs and diagnostics should be supplied according to the capacity and location of health facilities.
